# 

**Published:** 1865-03

**Authors:** 


### CONTENTS.

### ORIGINAL CONTRIBUTIONS.

ART. VII. Treatment of Uterine Tumors. By Wm. H. Byford, A.M., M.D., Prof. of Obstet., &c., in Chicago Medical College, ......... 129

ART. VIII. The Recent Investigations of Ricord and Others upon the subject of Syphilis. By E. Andrews, M.D., Prof. of Surgery in Chicago Medical College, ......... 133

ART. IX. Case of Acute Gastritis.—Sudden Death. By Hiram Nance, M.D., Kewanee, Illinois, ......... 139

ART. X. Cerebro-Spinal Meningitis. By F. R. Payne, M.D., Marshall, Illinois, ......... 143

ART. XI. A Thesis on Hospital Gangrene. By John E. Link. Presented to the Faculty of the Chicago Medical College, Feb. 1865, .. 147

### SELECTIONS.

On Remittent Ophthalmia—Strumous Ophthalmia of Children. By H. Hancock, Esq., Surgeon to the Charing-Cross and Westminster Ophthalmic Hospitals, &c., ......... 151

Review and Abstracts from a Collection of Memoirs upon an Important Discovery of some of the Unknown Functions of the Pancreas. By Prof. Lucien Corvisart, Physician to the Emperor, &c. Translated by J. K. Bauduy, M.D., St. Louis, Mo., ......... 156

Clinical Remarks on the Value of Belladonna in the Treatment of Iritis. By Jabez Hogg, Esq., Assistant-Surgeon Royal Westminster Ophthalmic Hospital, &c., ......... 165

On Treatment of Squint by Operation. By J. W. Hulke, Esq., Surgeon to the Royal London Ophthalmic Hospital, Moorfields, ......... 168

### BOOK NOTICES.

Proceedings of the American Pharmaceutical Association, at its 12th Annual Meeting, held in Cincinnati, Sept. 1864, ......... 171

Alphabetical Index to Braithwaite's Retrospect. Embracing Parts 1 to 50 —1840—1865, ......... 172

Recent Progress in Surgery. The Annual Address delivered before the Massachusetts Medical Society, May 25, '64. By J. M. Warren, M.D. 172

### EDITORIAL.

Annual Meeting of American Medical Association, ......... 172

Chicago Medical Society, ......... 172

New York Medical Journal, ......... 173

The Purification of the Press, ......... 173

Sulphuric Ether versus Chloroform. By J. F. Lente, M.D., ......... 176

Illinois State Medical Society, ......... 179

American Medical Association, ......... 180

Practical Items.—Specimen of Entozoa.—Therapeutics.—Turpentine in Chorea.—Removal of foreign bodies from the Nostrils, ......... 181

The Larynx of the Negro. By George D. Gibb, M.D., ......... 183

Report of the weights and measures used in Pharmacy. By B. S. Proctor, 185

The Black Troops of the British Army and Consumption, ......... 186

On the Internal Employment of Essence of Turpentine in the Headaches of Nervous Women, ......... 187

Substitutes for Indian Ink, ......... 188

Physiological Effects of Digitalis, ......... 188

Mortality of Chicago for March, 1865, ......... 189

Foreign Medical Intelligence, ......... 189

Demand for Physicians, ......... 190

